# Improvement of the combustion, emission, and stability features of diesel-methanol blends using n-decanol as cosolvent

**DOI:** 10.1038/s41598-022-20326-0

**Published:** 2022-11-08

**Authors:** Ahmed I. EL-Seesy, Mahmoud S. Waly, Alhassan Nasser, Radwan M. El-Zoheiry

**Affiliations:** 1 Mechanical Engineering Department, Benha Faculty of Engineering, Banha University, Benha, 13512 Egypt; 2 Chemical Engineering Department, Faculty of Engineering, Alexandria University, Alexandria, Egypt

**Keywords:** Mechanical engineering, Environmental impact

## Abstract

This research endeavored to boost the applicability of methanol in CI engines utilizing n-decanol as cosolvents. The work was split into binary phases. Firstly, the stabilities of pure methanol (*M100*) and hydrous-methanol (*MH10*), with diesel as a reference fuel, were examined applying various temperatures: 10 °C, 20 °C, and 30 °C. The findings showed that the *M100*-diesel and *MH10*-diesel combinations were unstable. Thus, n-decanol was utilized as a cosolvent. Following by the engine combustion and emissions characteristics were evaluated by manipulating three proportions of M100-diesel mixtures with n-decanol. Three mixtures comprised of 5, 10, and 15% M100 with 20% n-decanol, which are denoted as M5, M10, and M15, correspondingly. These combinations were assessed via thermogravimetric assessment, and their physicochemical properties were assessed corresponding to the ASTM. The maximum in-cylinder pressure, heat release rate, and pressure rise rate diminished by 10, 11, and 10%, respectively, for the M100/diesel/n-decanol combinations compared with the diesel oil. The brake thermal efficiency lowered by 10%, whereas the brake specific fuel consumption enlarged by 10% for the combinations compared with the diesel. NO_x_ and smoke opacity levels diminished by about 30 and 50%, respectively, whereas the CO and UHC enlarged by about 50 and 60% for the blends compared with the diesel oil.

## Introduction

The economic usability and availability of energy are key challenges influencing our daily life^1^. Consequently, the recognition of fitting alternative fuels for exploitation in burning systems, including compression ignition (CI) engines, is an imperative challenge^2^. Additionally, the utilization of petroleum oils drastically grows CO_2_ concentrations in the environment. In this respect, the application of fuels established from ecological sources, including biodiesel and alcohols, has been recommended to diminish combustion by-products risks^2,3^.

Precisely, methanol has been identified as a promising replacement fuel in the last years. This is attributed to its widespread suppliers, bulk manufacture, and decent physical and chemical assets^4^. Consequently, methanol is considered an alternative fuel that has grown to be one of the essential options for green burning in CI engines^5,6^. Nevertheless, some concerns are associated with manipulating methanol (M100), comprising the substitute path, cold initiation, ignition obstacle below part-load situations, and combustion fluctuation^7^. The highly essential aspect of unconventional fuel established for CI engines is its relevant cetane number (CN), which is small for M100^1^. Furthermore, the feasibility of M100 has been examined for centuries by combining it with diesel fuel attributing to its renewability and O_2_ substance, which would drastically drop the subsequent soot concentrations^7,8^. The quantity of NO_*x*_ created by M100 is also minimized owing to its elevated latent heat of vaporization (*LHV*) that drops the combustion temperature. Additional constraints of M100 as an alternative for diesel fuel comprise its less heating value (*HV*) and combination constancy concerns while it is mixed with fuel^9,10^.

Methanol (M100) can be used in CI engines using two techniques, including blended mode or dual mode. The blending mode includes the mixtures of M100 and diesel using emulsifiers or cosolvents^11^, while in the dual-mode, methanol is individually implanted into the inlet manifold^5,12^. The key benefit of mixing M100 and diesel fuel is that M100 is successively inserted into the combustion region and seems in zones, where it can substantially decrease pollutions. The addition of emulsifiers or cosolvents have been deemed a route to tackle the miscibility concern^5^. Limited kinds of research have assessed the effects of inserting M100 into an exhaust manifold to alleviate the cooling effect of M100^13^. Nour et al.^14^ examined the addition of ethanol and water in the exhaust manifold to eliminate their endothermic consequences. They reported that there was an increase in peak in-cylinder pressure, mean effective pressure, and ignition delay for ethanol/water blends compared to diesel.

Venu and Madhavan^15^ examined the engine performance using diesel/biodiesel/M100 (20% by volume) and diesel/biodiesel/ ethanol (20% by volume) blends utilizing diethyl ether (DEE) as additives. They stated that adding DEE to the mixtures increased the combustion duration, in-cylinder pressure, and brake-specific fuel consumption while the NO_x_, PM, and smoke levels lowered. They suggested that this might be accredited to the drop in the ignition delay and elevated *LHV* of the M100 with adding DEE. Sayin^16^ researched the burning and emissions factors of a diesel engine running with diesel/ M100 (5,10, and 15% by volume) blends with varying injection pressure and length. The experiments were performed at injection pressures of 180, 200, and 220 bar and timings of 15, 20, and 25 deg.BTDC. They indicated that the increased M100 fraction in the mixtures reduced the BSFC, smoke opacity, CO, and total unburned hydrocarbon (THC), while the NO_x_ level increased. They stated that there was no positive consequence on the engine performance with changing injection timing and pressure.

Another study by Jiao et al.^17^ has investigated the combustion and emission characteristics of a diesel engine driven by a diesel/biodiesel/ M100 (15.2% by volume) blend. They also studied this blend at different altitudes of zero m, 3500 m, and 5500 m. They stated that the in-cylinder pressure, dp/dtheta, and HRR reduced for fuels combinations compared to diesel, at the elevation of zero m. However, the opposite trend recorded at the height of 5500 m. Hasan et al.^18^ explored the consequences of implanting methanol (10, 20, 30, and 40% by volume) with diesel fuel on the combustion and emission aspects of a CI engine. They reported that the BTE reduced, and BSFC increased for fuel combinations compared to diesel fuel. They also declared that the NO_x_ level was high for the combinations compared to diesel fuel, while CO, UHC, and soot levels slumped^19^.

Jamrozik^20^ studied the impacts of adding M100 (10, 20, 30, and 40%) to diesel fuel on engine performance. They mentioned that the BTE enhanced with adding M100 up to 30%; after that, the COV increased by over 10%. They also reported that the CO level reduced, and NO_x_ level increased, while the UHC unchanged with adding methanol. They stated that the in-cylinder pressure intensified, and their peak places retarded. This was due to the increase in the ignition delay. Yilmaz^21^ scrutinized the combustion and emission characteristics of a CI engine driven by diesel/biodiesel/M100 (10% by volume) and diesel/biodiesel/ethanol (10% by volume) blends. They reported that the CO and UHC concentrations increased with enlarging the M100 or ethanol in the mixtures, while NO_x_ levels lowered. They mentioned that M100 combinations were more efficient than ethanol combinations for lowering CO and UHC levels, while NO_x_ level reduced with applying ethanol mixture.

Chen et al.^22^ scrutinized the miscibility of M100/diesel combinations applying n-pentanol as a cosolvent under various temperatures. Then, they assessed their combinations (10 and 15% of M100) on the combustion and emission aspects of a CI engine. They mentioned that using n-pentanol as a cosolvent established homogenous mixtures of methanol and diesel under distinct temperatures. They also described that the ignition delay lengthened, combustion duration reduced, and the highest combustion temperature soared with implanting methanol. Also, the soot level reduced, while NO_x_ level increased with inserting M100. Mariappan et al.^23^ optimized the production of bi-oil from pyrolysis plastic oil and then assessed the combustion and emission aspects of a CI engine driven by bio-oil/D100 blends and bi-oil/M100 (15 and 20%)/ diethyl ether mixtures. They mentioned that the addition of M100 instigated a reduction in the CO, UHC, soot levels, while the NO_x_ concentration increased. They also reported that the BTE reduced with adding methanol. Zhang et al.^24^ examined experimentally and numerically the effects of adding n-butanol to diesel-methanol blends on engine performance. They reported that the fuel spray and combustion processes significantly affected by diesel/methanol/n-butanol blends. They mentioned that the addition of methanol to diesel fuel resulted in an increase in ignition delay, in-cylinder pressure, and peak heat release rate compared with diesel. They also recorded that NO_x_, CO, soot, and HC levels reduced for diesel-methanol-n-butanol blends compared to diesel. They noticed that the recommended blending ratio was 70% diesel + 20% methanol + 10% n-butanol. The same authors have examined the combustion and emission aspects of a diesel engine working with diesel-ethanol-n-butanol blends^25^. They reported that brake thermal efficiencies and brake specific fuel consumption increased, while NOx, CO, and soot reduced for diesel-ethanol blends compared with diesel.

Sayin et al.^26^ assessed the engine performance run with diesel/methanol (5, 10, and 15% by volume) blends with various injection timings. They reported that the BTE, smoke opacity, and UHC levels lowered with enlarging the proportion of methanol in the blends. They stated that the NO_x_ level diminished, and smoke opacity, UHC, and CO levels enlarged for the delayed injection timing compared to the original timing. They also asserted that smoke opacity, UHC, and CO lowered, and NO_x_ enlarged with applying the advanced injection timing. They declared that the BTE and BSFC had a good trend at original timing. Fan et al.^27^ evaluated the combustion and emission factors of a diesel engine working with diesel/methanol (13%), manipulating dodecanol and nitric acid ester as dissolvability improvers. They reported that the BTE reduced for the mixture comparison with diesel fuel. They also stated that the fuel mixtures generated a high level of formaldehyde, acetaldehyde, acrolein, acetone and crotonaldehyde compared to diesel fuel, while it reduced propionaldehyde level. A related study by Hassan et al.^28^ manipulated the dodecanol to heighten the miscibility issue of diesel/methanol (7, 14, and 21%) blends. They stated that there were sizeable augmentations in combustion and emission factors with implanting methanol.

Yasin et al.^29^ researched the combustion and emission quality of a CI engine run with diesel/biodiesel/methanol (5% and 10%) mixtures. They mentioned that the bsfc for the mixtures was smaller than diesel fuel, while NO_x_ increased, and CO lowered. Verma et al.^10^ assessed the engine performance run with ethanol (20%)/methanol(20%)/diesel/microalgae mixtures. They mentioned that the in-cylinder pressure increased for the mixture compared to diesel fuel. The CO, UHC, and soot levels lowered, while NO_x_ enlarged for the fuel mixtures.

Higher alcohols (C_4_ to *C10*) are believed to be utterly miscible with both diesel and biodiesel fuels^30^, and may possibly recompense for variations in the fuel aspects, which would boost the whole burning efficiency and emission concentrations. Thus, academics have commenced considerable attempts to augment alcohol application to achieve an enriched fuel with oxygen by examining the engine performance of D100-higher alcohol combinations^13,31^. It is recognized that higher alcohols have convinced motivating features, comprising their usage as enriched fuels with oxygen and cosolvents for enhancing the utilization of M100 in CI engines and other sectors. These features can be used to moderately tackle the issues practiced in the application of lower alcohols as fuels^32^. Additionally, higher alcohols are described by elevated *CN* and *CV* than those of M100^33^. Also, their *LHV* is lower than that of lower alcohols^34^.

Moreover, n-decanol is demonstrably colorless to slightly yellow fluid, carrying ten carbon atoms in its structure. It is applied largely in nutrition and substance production than as a replacement fuel for CI engines^35^. However, coconut manipulation as a supplier for n-decanol production is augmented, which contains decanoic acid-like Yarrowia lipolytica, and the creation cycles are perceived to be economically affordable with a massive yield^36^. Thus, the capability for utilizing botanical biomass and bio-supplies containing lignocellulosic material and waste protein has convinced the researchers to evaluate n-decanol as a practical fuel. Moreover, it holds a greater heating value than the bulk of the current biodiesels and its other alcohol family. Its boiling point meets the range of diesel boiling trends, and it does not comprise any aromatic structures. These attractive features make it a good candidate to be used as a cosolvent in the current study.

The manipulations of implanting the n-decanol with diesel/biodiesel mixtures on the CI engine performance are collected in Table 1. These investigations have clarified that the bsfc enlarged, as well as soot concentration slumped, while the P_cyl._, HRR, and ID, as well as UHC, CO, and NO_x_ concentrations depended on the engine operating condiations^37,38^. Additionally, there are limited studies that have evaluated the effects of incorporating n-decanol with pure diesel/M100 blends.

**Table 1:** Combustion and emission characteristics of CI engines driven by n-decanol/diesel/biodiesel combinations.

Engine	Base fuel	Running situations	Alcohol kind	M100 Fractions	Performance findings	Combustion findings	Emission findings	Reference
One Cylinder, 661 cc	Diesel/biodiesel	Fixed speed with differing loads	n-Decanol	5, 10, 15, 20, 30 and 40% vol	Augmented BTE	Augmented p, HRR and ID	Dropped NO_x_, UHC, CO and soot	^39^
One Cylinder, 661 cc	Diesel/biodiesel	Fixed speed with differing loads	Hexanol and *n*-Decanol	30 and 40% vol	Inflated BTE	Augmented p, HRR and ID	Dropped soot, NO_x_, UHC and CO	^40^
One Cylinder, 661 cc	Diesel/biodiesel	Fixed speed with differing loads	n-Decanol	10% vol	Enlarged BTE, lowered BSFC	Augmented p, HRR and ID	Dropped soot, UHC, and CO; increased NO_x_,	^41^
One Cylinder, 661 cc	Diesel/biodiesel	Fixed speed with differing loads; varied injection timings with EGR	n-Decanol	10, 20, and 30% vol	Enlarged BTE; lowered BSFC	Lowered p, HRR and ID	Dropped soot, UHC, and CO; increased NO_x_,	^42^

### Study aims and motivations

Methanol (M100) is proposed to be the most favored enriched fuel with oxygen for diesel engines attributed to its exceptional fuel characteristics, involving plentiful ecological resources, accessibility, and being reasonably priced. Nevertheless, the applications of M100 in CI engines have some issues counting: (1) miscibility problem (accredited to the M100 holds a percentage of water), (2) small CN (reasons struggle in launching combustion and increased ID), (3) elevated latent heat of vaporization (*LHV*) (generates a quenching effect throughout combustion), and (4) small heating value (lowered BTE)^43–45^.

To tackle the miscibility matter of M100 with diesel oil, n-decanol was utilized as a cosolvent. It was nominated depending on its appropriate aspects of elevated miscibility with M100 and diesel fuel, small *LHV*, and elevated heating value compared with M100. It is worth mentioning that several kinds of higher alcohols, such as n-hexanol, n-heptanol, n-octanol, and n-decanol, have been pretested as cosolvents to alleviate the phase stability issue for methanol-diesel mixtures, and the result illustrated that n-decanol gave a promising capability as a cosolvent.

Nevertheless, to the most excellent of our experience, no research has tested the miscibility issue of M100/hydrous methanol /diesel combinations. Furthermore, the influences of utilizing n-decanol as a cosolvent with M100/diesel mixtures have not been assessed. Accordingly, to meet this examination gap, the purposes of the existing work were to: (1) examine the miscibility issue of M100/hydrous methanol/diesel combinations utilizing n-decanol as a cosolvent at respective temperatures (10, 20, and 30 °C); and (2) evaluate the capability of n-decanol as cosolvents to augment the miscibility matter and ignition quality of methanol/diesel combinations. Hence, 5, 10, and 15 vol% of M100 were mixed with 20% n-decanol with diesel oil as the reference fuel, which were implied as M5, M10, and M15, correspondingly.

## Materials and approaches

### Miscibility assessment

In this part, n-decanol was applied as a cosolvent to augment the mixture solidity of M100/diesel blends and hydrous methanol/diesel combinations at various temperatures of 10, 20 and 30 ºC. n-decanol was manipulated as a cosolvent after assessing several kinds of higher alcohols (*C3*–*C10*). The n-decanol was selected as a cosolvent after several tests of different kinds of higher alcohols, such as propanol, butanol, pentanol, and n-decanol. It showed high potential to solve the issue of phase separation of methanol with diesel fuel.

**Figure 1:**
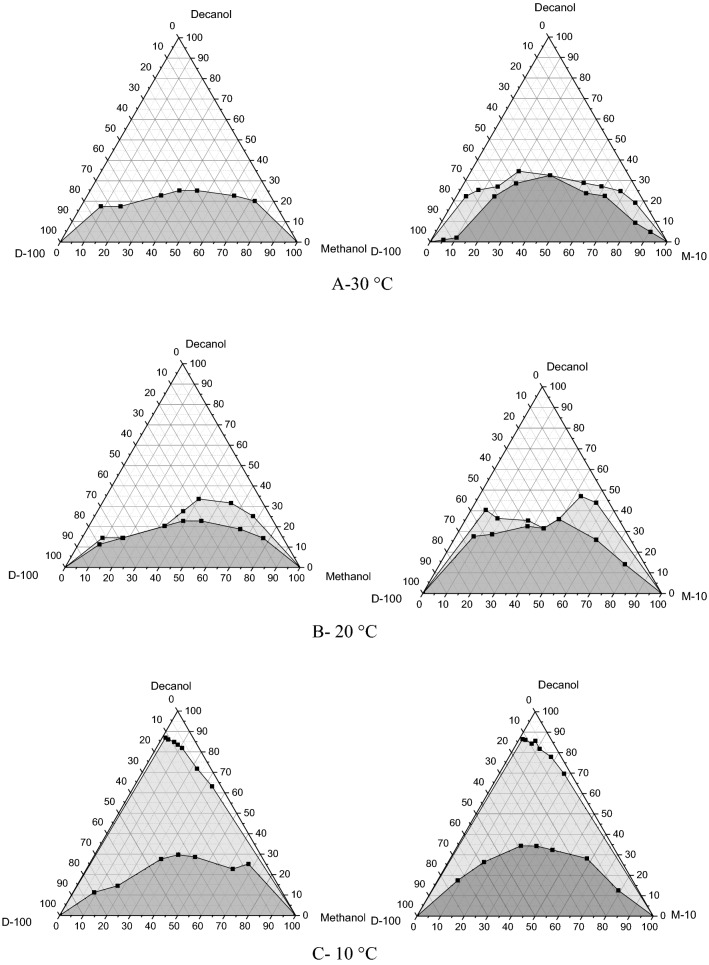
Change in the miscibility of M100 and hydrous methanol with diesel, manipulating n-decanol as cosolvent, at several temperatures.

To formulate the hydrous methanol/diesel combinations, 10 ml of hydrous methanol (MH10 = 90 wt.% M100 and 10 wt.% purified water combination) dual combinations were formulated in numerous concentrations varied from 0 to 100 vol% with a 10% increase. These combinations were primarily in dual-region equilibrium attributing to the immiscibility of pure methanol and MH10 in the diesel fuel. Then, they were measured accurately with the n-decanol as the titrant utilizing very accurate 1-ml tubes, and the termination at which the combination established a one consistent appearance was verified as a zone sitting on the border of the triple-stage figure. The terminations evaluated for every test were utilized to create the triple stage graphs in mass proportion for evaluating the triple combination stage performance, as illustrated in Fig. 1. This experiment principally concentrated on the disappearance of the dual-stage border; the stage trend at elevated dose levels of cosolvent was not investigated. Corresponding to published studies, it could create gel stages, and such combinations are not applicable as fuel^9,46^.

In certain situations, the phase of disappearance was not strong as there was a conversion stage among the dual-stage and single-stage manners in which a gloomy stage seemed prior to a transparent mixture was achieved upon additional accumulation of the cosolvent. This gloomy zone was also mentioned in the segment graphs, as demonstrated in Fig. 1. The stage trend of the pure methanol/diesel and hydrous methanol/diesel mixtures was examined at temperatures of 10 °C, 20 °C, and 30 °C. It is noticeable that the temperature substantially affected the stage solidity of the mixtures. Furthermore, the portion of n-decanol needed to accomplish the uniform level was enlarged by decreasing the combination temperature. This trend might be ascribed to that the thermal flow boosts as the temperature increases, which is favorable to the diffusion and dissemination of molecules^9^. Equivalent findings were stated by Liu et al.^9^. To assess the phase stability performance for each blend, all trials have flowed into a lengthy glass cylinder for almost 60 days, and there was no phase separation detected.

### Diesel engine setup

**Figure 2:**
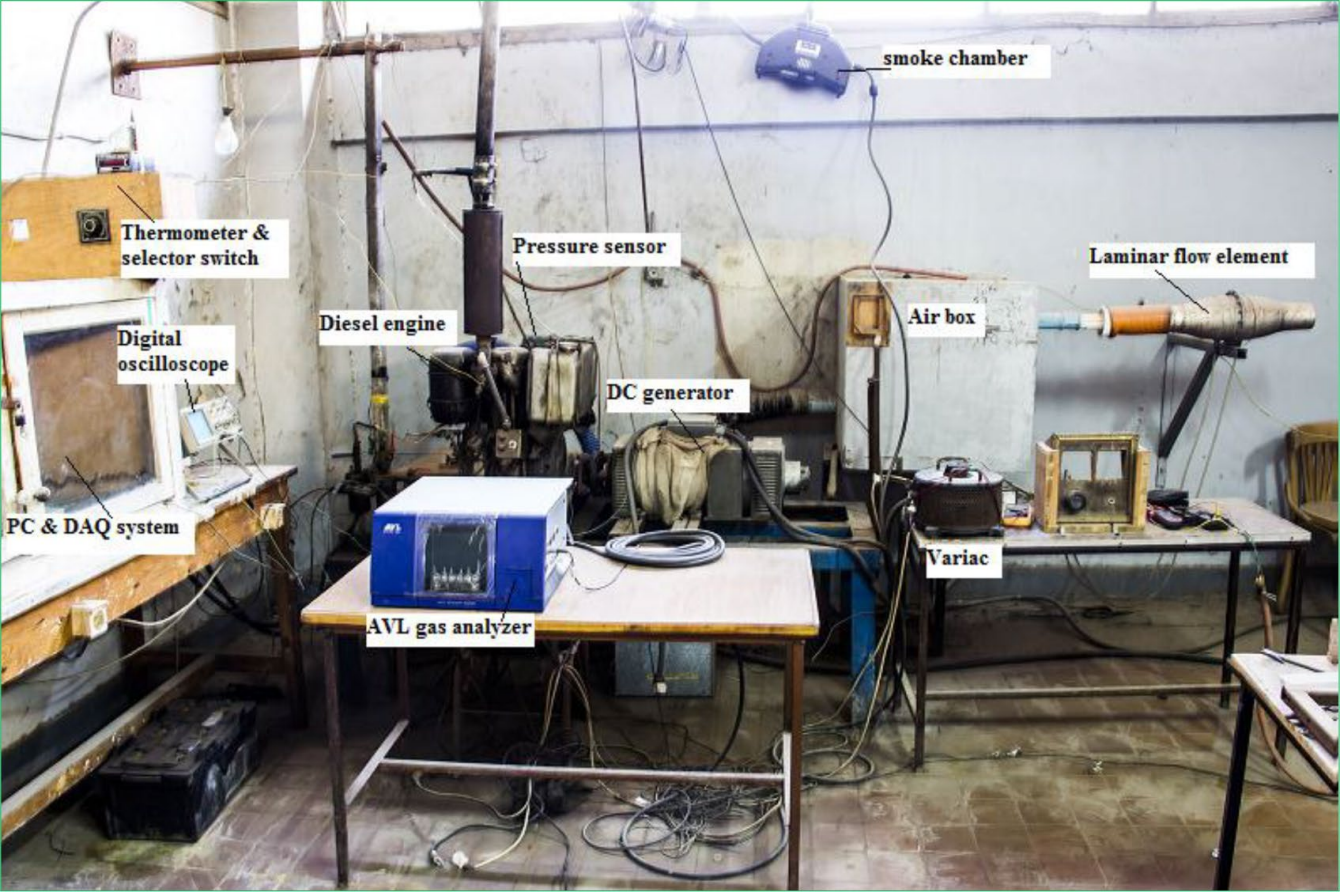
Photo of the test rig.

The experimental scheme involved a diesel engine feature, in-cylinder pressure recorded scheme, engine performance assessment technique, and emission evaluation devices. The outline is displayed in Fig. 2. Explanations of the manipulated devices can be found in article^47^.

Moreover, for each individually tested state, the in-cylinder pressure (*P*_*cyl.*_) information were logged for 50 repeated cycles and averaged to get the fitting *P*_*cyl.*_, which was applied to assess the HRR. The estimation of the cyclic alterations also disclosed acceptable constancy in the engine, ascribing to the coefficient of variation for all assessed situations was smaller than 4%^48^. Additional data on the test facility can be achieved in the literature^49^. The HRR was assessed applying the subsequent formula^49^:

1 $$\frac{{dQ_{gross} }}{d\theta } = \frac{{\gamma_{(T)} }}{{\gamma_{(T)} - 1}}*p*\frac{dV}{{d\theta }} + \frac{1}{{\gamma_{(T)} - 1}}*V*\frac{dp}{{d\theta }} + \frac{{dQ_{wall} }}{d\theta },$$

where γ_(T)_ of the gases was calculated using the following equation^50^:

2 $$\gamma_{(T)} = 1.35 - 6*10^{ - 5} *T + 10^{ - 8} *T^{2}.$$

Using the recorded in-cylinder pressure and the computed cylinder volume, the gas temperature *T* could be computed using the equation of state as follows^51^:

3 $$T = \frac{{T_{r} *p*V}}{{p_{r} *V_{r} }}$$

All thermodynamic states (pressure (*p*_r_), temperature (*T*_r_), and volume (*V*_r_)) were investigated at a given reference situation, such as IVC (*p*_IVC_, *T*_IVC_, *V*_IVC_). *T*_IVC_ and *p*_IVC_ are the temperature and pressure at the IVC, corresponding to 350 K and 1.013 × 10^5^ Pa, respectively.

The rate of heat transfer from the gases to the cylinder wall was computed using the convection heat transfer equation as follows^52^:

4 $$\frac{{dQ_{wall} }}{d\theta } = h_{c} *A_{(\theta )} *(T - T_{wall} )*\left( \frac{1}{6N} \right).$$

The heat transfer calculations were not sensitive to the wall temperature *T*_w_. The wall temperature of 450 K was anticipated and found to provide acceptable findings^51^. The instantaneous combustion chamber surface area *A*_(*θ*)_ includes many surface areas, as follows^52^:

5 $$A_{cy(\theta )} = A_{ch} + A_{pc} + A_{lat(\theta )} .$$

For flat-topped pistons, *A*_pc_ = (*π*/4) × *B*^2^. The lateral surface area *A*_lat(*θ*)_ approached the lateral surface of the cylinder, and *A*_ch_ was assumed to be equal to *A*_pc_. Consequently, the instantaneous combustion chamber surface area was computed using the following equation:

6 $$A_{cy(\theta )} = \frac{{\pi *B^{2} }}{4} + \frac{\pi *B*L}{2}*(R + 1 - \cos (\theta ) - (R^{2} - \sin (\theta )^{2} )^{0.5} ).$$

There are numerous models for the heat transfer coefficient *h*_c_. In addition, there are numerous studies that recommend using the Hohonberg correlation for diesel engine combustion analysis to assess the convection heat transfer coefficient^53,54^. This requires simple calculations that provide precise findings instantaneously. Thus, the following correlation was utilized to assess the convection heat transfer coefficient^55^:

7 $$h_{c} = C_{1} *V^{ - 0.06} *p^{0.8} *T^{0.4} *(C_{2} + V_{m} )^{0.8}$$

where *p* is the instantaneous pressure in the bar. The numerical values *C*_1_ = 130 and C_2_ = 1.4, as shown in the above equation, are constants formed by six representative engines.

### Fuel preparation and assessment

In this study, diesel fuel was utilized as the reference fuel from a regional supplier. The methanol and n-decanol used in this experiment had purities of 99.9%. Three mixtures of methanol/diesel/n-decanol were utilized for engine evaluation, as scheduled in Table 2. The assessed fuels are signified as M5, M10, and M15, correspondingly. The methanol/diesel/n-decanol mixtures were mixed by applying an ultrasonic instrument (Hielscher), which was modified at the rate of 24 kHz for almost 5 min for every blend. No miscibility issue was identified for the assessed mixtures through or following the experiment. The physicochemical aspects of diesel, M100, and n-decanol are demonstrated in Table 3.

**Table 2:** Test environments.

Fuel	Working terms	Injection situation	Assessed aspects
1. Diesel (D100)	BMEP = 0, 1.5 bar (25%), BMEP = 3 bar (50%) , BMEP = 4.5 bar (75% load); and 1,500 rpm	SOI: 24°bTDC IP: 175 bar	1. P-theta, HRR, dp/dtheta, X_b_, ID, and CD
2. 5% methanol + 20% n-decanol + 75% D100 (M5)			2. BTE, BMEP, BSFC, EGT, BSEC, and equivalence ratio
3. 10% methanol + 20% n-decanol + 70% D100 (M10)			
4. 15% methanol + 20% n-decanol + 65% D100 (M15)			3. Smoke opacity [%], NO_*x*_ [ppm], UHC (ppm) and CO [ppm]

**Table 3:** Properties aspects of D100, methanol, and n-decanol^16,39,42^.

Property	Diesel	M100	n-decanol
Molecular formula	C_*x*_H_*y*_	CH_3_OH	C_10_H_21_OH
Molecular weight (g/mol)	211	32.04	158.23
Density (kg/m^3^) at 15 °C	835	796	829
Boiling point (°C)	180–360	64.5	233
Carbon (wt.%)	86.1	–	68.23
Hydrogen (wt.%)	13.9	–	12.64
Oxygen (wt.%)	0	50	10.11
CN	48	3	50
Viscosity at 40 °C (mm^2^/s)	2.7	0.59	6.5
HV (MJ/kg)	42.5	19.9	41.82
LHV (kJ/kg)	270	1,109	494.8

**Figure 3:**
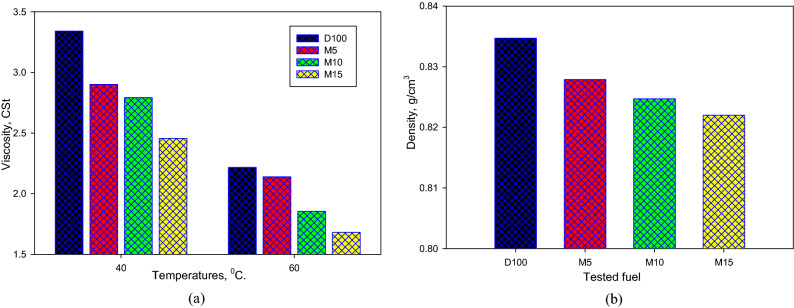
(**a**) Viscosities and (**b**) densities of assessed mixtures.

The change in viscosity against temperature for the assessed fuels, evaluated corresponding to the ASTM standard, is demonstrated in Fig. 3a. It can be perceived that the viscosities of the M5, M10, and M15 mixtures are smaller than that of diesel fuel. The viscosity decreased by almost 13%, 16%, and 26% at 40 °C, and by about 4%, 16%, and 24% at 60 °C for the M5, M10, and M15 combinations, correspondingly. Figure 3b depicts the variation in density for the assessed fuels, evaluated corresponding to the ASTM standard. The densities of the M5, M10, and M15 mixtures are smaller than that of the D100.

**Figure 4:**
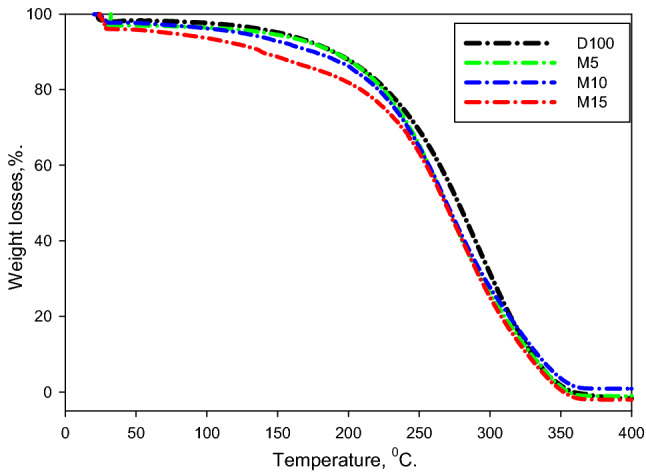
TGA assessment for evaluated fuels.

Figure 4 indicates the change in TGA assessment for the assessed fuels, which are evaluated applying Setaram LABSYS EVO. The findings imply the fuel-mass ratio reduced with growing the temperature, which would help to understand the fuel combination developments during fuel evaporation. As the temperature enlarged, the evaporated portion of the multi-compound fuel enlarged, and hence the specimen mass decreased until the entire fuel decomposed^56^. It is remarked that the diesel fuel started evaporating at 150 °C and ended up evaporating at 365 °C. It can also be noticed that the M5 and M10 mixtures began to evaporate at about 120 °C and 100 °C, correspondingly, which was finished at almost 350 °C. At the same time, the M15 combination began to evaporate at about 60 °C and completed evaporating at 345 °C. It is noticeable that boiling was started promptly for the fuel combinations. Furthermore, the evaporation ratio for the fuel combinations improved as the M100 portion in the mixtures increased.

In this experiment, the mixtures were examined below thermally steadied stable-state environments, subsequent the procedures and proposals in article^49^. The uncertainties for both the assessed and calculated factors were expected by utilizing the Holman technique^57^, as presented in Table 4.

**Table 4:** Uncertainty assessment.

Gauged aspect	Uncertainty
Burning pressure	± 1% FSO
Air volume flow rate	± 1% FSO
Differential pressure	± 0.1% FSO
Fuel volumetric flow	± 0.1 mL
Temperature	± 1 °C
Rotational speed	0.2% FSO
Smoke opacity	0.1% FSO
CO	0.1% FSO
NO_*x*_	0.2% FSO
BSFC	1.4% FSO
BP	1.1% FSO

## Findings and discussions

### Combustion assessment

**Figure 5:**
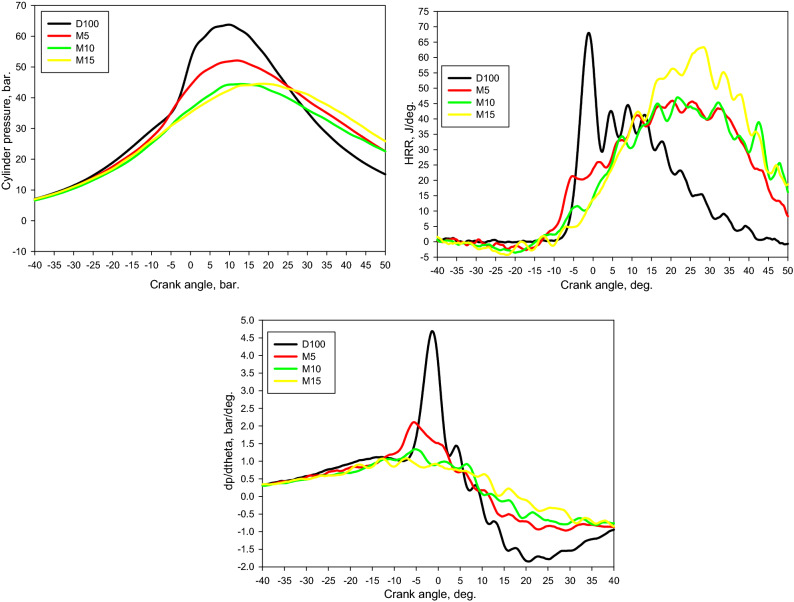
Discrepancy of *P*_*cyl.*_, HRR, and dp/dtheta via crank angle for tested fuels at bmep of 4.5 bar.

Figure 5 shows the discrepancies in the in-cylinder pressure (*P*_cyl_), heat release rate (HRR), and pressure rise rate (d*p*/d*θ*) against the crank angle. Remarkably, the combustion development appeared to shift from mixed-controlled diffusion-burning (typical diesel-burning) for diesel to kinetically controlled burning for the methanol mixtures, which is similar to the combustion mode in modern CI engines. It is noticeable that the HRRs of D100 demonstrate the standard figure of diesel combustion (four phases: ignition delay, premixed phase, main diffusion phase, and late burning phase). On the other hand, the methanol mixtures validate an HRR shape representative of burning processes dominated by a progressive autoignition occurrence. This performance would also clarify the reduction in soot levels of the methanol combinations, as depicted in “Engine emissions” section.

In addition, it is noticeable that the M5, M10, and M15 mixtures had lower *P*_cyl_, HRR, and d*p*/d*θ* compared to D100; where they lowered on average by 10%, 11%, and 10%, respectively for M5, M10, and M15 blends. Moreover, there are two peaks detected in the HRR of the mixtures, the peaks of which enlarged with enlarging a portion of M100 in the mixture, and their locations were delayed. The ternary combinations have distinctive characteristics, including high CN, small viscosity, small HV, small density, large O_2_ ratio, and superior latent heat of vaporization (LHV). Nevertheless, the LHV regulates the burning developments. This may possibly be ascribed to the fact that methanol includes a higher LHV, prompting a quenching effect in the combination. This consequence enlarged with enlarging methanol portion. The TGA findings emphasize these outcomes; the evaporation level was diminished by expanding the methanol segment in the combination (Fig. 4). Consequently, the impacts of the quenching consequence from implanting methanol on the mixtures were marked. These results are similar to those depicted in formerly available studies^22,58^.

**Figure 6:**
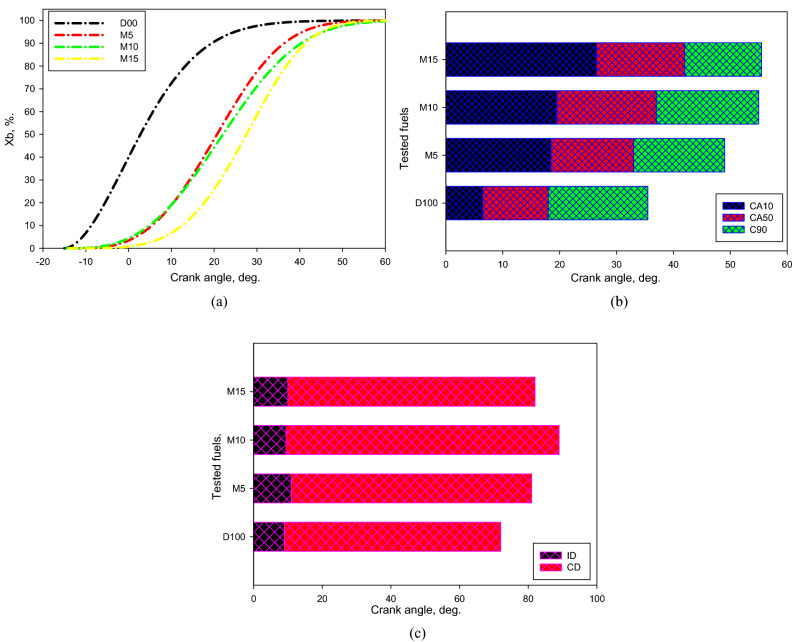
Discrepancy of X_b_, CA10, CA50, CA90, ID, and CD via crank angle for tested fuels at bmep of 4.5 bar.

Figure 6 shows the differences in the mass burned fraction (X_b_), CA10, CA50, CA90, ID, and CD for the evaluated fuels. CA50 is an indispensable highlight for the combustion and emission features of a CI engine, and pinpoints the achievement of the premixed combustion area and the launch of the diffusion combustion region. The discrepancy among CA10 and CA50 predicts as a premixed combustion interval^52^. CA90 proves the end of combustion, and the discrepancy between CA10 and CA90 denotes the combustion phase. CA50 increases for the M5, M10, and M15 mixtures, and it enlarges with enlarging quantity of methanol in the mixtures. This may perhaps be ascribed to that methanol holds a raised LHV, triggering a quenching impact in the combinations and postponed combustion. Consequently, the combination burning appeared primarily in the diffusion approach.We suggested that the combination combusts in a nonuniform approach. This implies that the combustion is introduced by exceedingly volatile fuel, but the created energy was insufficient to evaporate all the M100. Hence, we deduced that the combination was primarily combusted in the diffusion approach. It can be remarked that the burning intervals were longer for the M5, M10, and M15 combinations comparison with that of the D100. This might be recognized by the raised LHV in these combinations.

The ID phase is characterized as the period interval, considered since the beginning of the injected fuel (24°.bTDC) to the SOC. The SOC is quite challenging to characterize, but it can be assessed by applying one or extra of the subsequent methodologies^59^. The SOC can be characterized as the position: (1) someplace the smallest PRR point appears in the initial derivative next to the beginning of injection on differentiating the *P*_*cyl*_ against the CA; (2) someplace the HRR grows to be zero. In the existing work, dual methodologies for the period postpone phase were assessed to get preferable outcomes. The principal estimation confirmed that the ID phase anticipated utilizing the PRR was equivalent to the HRR technique assessed. Hence, the ID phases for the various mixtures and working situations were assessed utilizing the HRR methodology. The ID and CD increased for M5, M10, and M15 blends compared with D100 fuel, as given in Fig. 6c. This could be attributed to the small cetane number of methanol and elevated latent heat of vaporization that could generate a quenching effect throughout the combustion developments.

**Figure 7:**
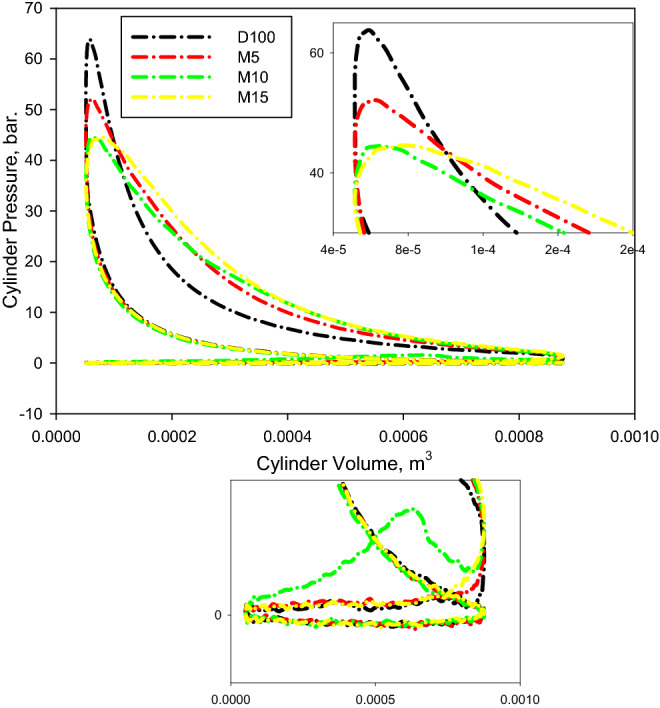
Change of *P*_*cyl.*_ vis *V*_*cyl.*_ at bmep of 4.5 bar.

Figure 7 indicates the change of P–V for the assessed fuels. It delivers a clean methodology to evaluate the net and attained work. The segment controlled by the boundaries demonstrating cycle practices characterizes the net-work supplied by the cycle. The area outlined in the picture for D100 is greater than those for the diesel/M100/n-decanol combinations. The pumping work of the fuel mixtures was noticed to be nearly equivalent to that of the D100.

### BSFC, BTE, EGT, and BSEC

**Figure 8:**
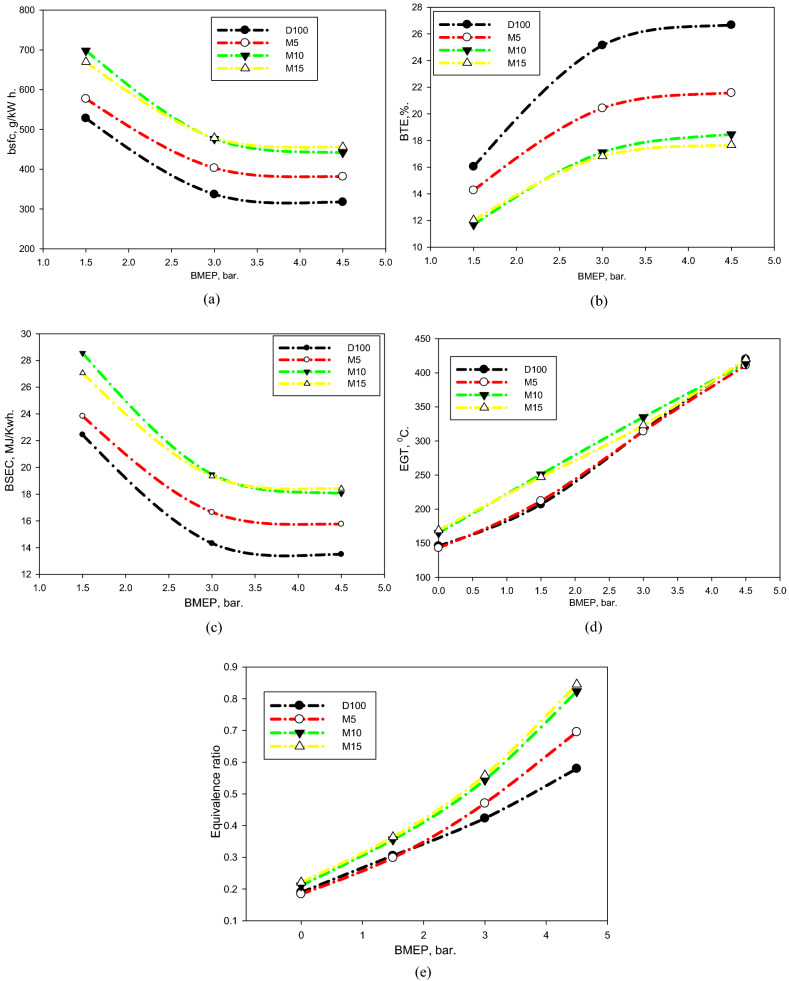
(**a**) BSFC, (**b**) BTE, (**c**) BSEC, (**d**) EGT, and (**e**) equivalence ratio of assessed fuels at different BMEPs.

The brake thermal efficiency (BTE) and brake specific fuel consumption (BSFC) for the evaluated fuels were drawn versus bmep, as illustrated in Fig. 8a, b. BSFC is primarily encouraged by the combustion efficiency of the fuel, wherever the exceptional burning highlight triggers a drop in it^60^. The BSFC increased for the M5, M10, and M15 mixtures comparison with D100. The fuel combinations have low HV, high density, low viscosity, and low CN comparison with those of D100. These exceptional characteristics might negatively impact the spray and burning development, initiating a worsening in the fuel evaporation phase, air/fuel mixing, combustion efficiency, and hence increased the BSFC. Moreover, the elevated LHV of methanol might regulate the combustion growth, in particular with a large volume of it in the combination that might trigger a reduction in the combustion efficiency^61^. An equivalent tendency is detected for the BTE for the evaluated fuels. These conclusions correlate to those illustrated in available studies^58,62^.

The brake specific energy consumption (BSEC) can be characterized as the energy conversion production to provide energy in MJ/kWh, and delivers a corresponding tendency of the BSFC^63^. The discrepancy in the BSEC for the tested mixtures via BMEP is demonstrated in Fig. 8c. It is realized that the BSEC tendencies are equivalent to the fuel combinations. Moreover, the fuel mixtures have different energy insides, which characterize the individual values of fuel use and delivered energy.

The difference in the EGT versus BMEP for the assessed fuels is displayed in Fig. 8d. From this picture, the EGT for fuel combinations was higher than that for D100. This might be clarified by the reduced energy contained by the combinations, as considered previously. These outcomes relate to those described in available studies^58^.

The discrepancy in the equivalence ratio (*ER*) versus the BMEP for the assessed fuels is displayed in Fig. 8e. The fuel capability improved with the disparity in the BMEP, which rectified the *ER*. Further fuel was presented through the increasing BMEP, and the whole *ER* fluctuated from 0.2 to 0.8. As the *ER* heightened, the burning temperature heightened, hence growing the ID^59^. Thus, the *P*_*cyl.*_ increased gradually with an intensifying *ER*. An escalation in the ER is noticeable for the combinations comparison with the D100. These outcomes are supported by the worsening of the BTE, and the growth in CO intensity, as will be debated in the subsequent paragraph.

### Engine emissions

**Figure 9:**
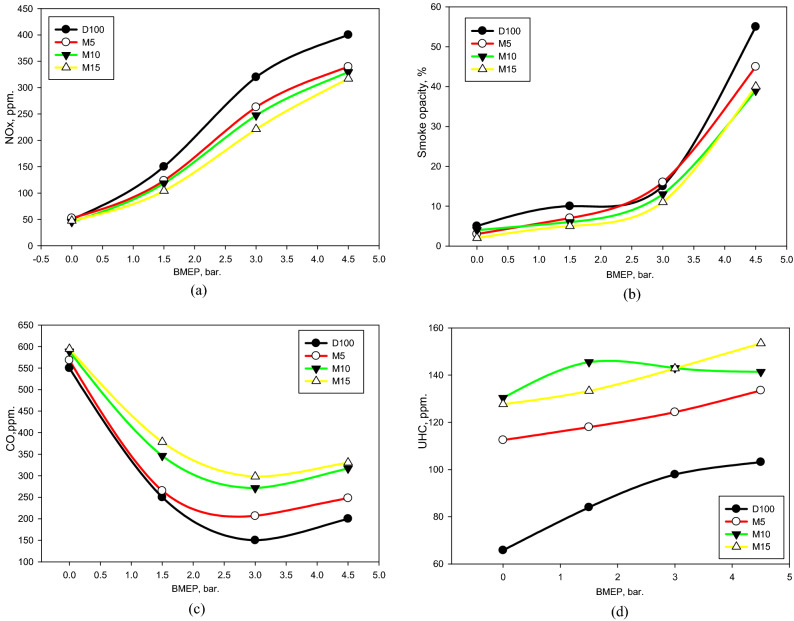
(**a**) NO_x_, (**b**) smoke opacity, (**c**) CO, and (**d**) UHC of assessed fuels at different BMEPs.

The variation in NO_*x*_ intensity with BMEP for the assessed fuels is demonstrated in Fig. 9a. It is noticeable that the NO_*x*_ intensity grows with the growing engine load. This might be ascribed to that the NO_*x*_ concentration is remarkably dominated by the combustion velocity, and dropped combusted speeds with lean patterns express a more lengthy period to create^64^. Comparably, chemical kinetics substantiates that NO_*x*_ development strengthens markedly with growing combust temperatures^64^. Hence, NO_*x*_ strengthened with an enlargement in BMEP. The creation of NO_*x*_ predominantly influences the temperature, locale intensity of O_2_, and the time of combustion^52,64^. Thus, it is produced within the diffusion-controlled combustion interval on the precise boundary of the reacting section^64^. Two endeavors have been manipulated to lower the NO_*x*_ amount by lowering the combusted temperature and declining the combusted time^64^. The NO_*x*_ amounts for M5, M10, and M15 mixtures are lower than that for D100. This might be credited to the incorporating of methanol, which holds a high *LHV* that creates the quenching influence of the combination and supports in diminishing NO_x_. Moreover, it is noticeable that M15 documented the lowest amount of NO_x_ pattern. We hypothesized that there was no sufficient phase to produce NO_x_, hence that it was diminished. This was validated by the decline in the burning interval of M15, as demonstrated in Fig. 6. These findings are reliable with those asserted in the articles^10,22,58^.

In addition, the smoke opacity escalations with an escalation in the load, as indicated in Fig. 9b. This might be anticipated to the progress of the incorporated fuel burned primarily via a diffusion structure. Smoke development arises at the fuel-rich boundary of the reacting region during the diffusion burn^64^. Through fuel decay from dense to soft hydrocarbons, soot atoms are established, grouped, and emitted with a high amount in the state of small O_2_ and small regional temperature. On the other hand, the formed soot atoms are converted to CO_2_, and their intensity is dropped in the emissions. Moreover, smoke intensity progression is correlated with the equivalence ratio (*ER*). This implies that when the *ER* achieves the stoichiometric level, there is a small availability of O_2_ to convert all the soot established inside the reacting area, even as the cylinder temperature is elevated. It is noticeable that the smoke opacity is lowered for M5, M10, and M15 mixtures compared with the D100. Although there is also an opposite relation among NO_x_ and smoke opacity^10^, the unique aspects of the mixture, particularly oxygen enrichment, could facilitate reducing soot levels. Remarkably, M15 had the lowest amount of soot growth. This may possibly be anticipated to the boosted O_2_ percentage in the combination with escalating M100 segment.

The variation in CO levels for the mixtures via BMEP is displayed in Fig. 9c. It is noticeable that the CO amount declines with the increase in the BMEP, barring at elevated BMEP. This might be ascribed to the growth in the intensity of the oxidation mechanism of CO with the increase in combustion temperature [75]. The CO amount is elevated for the M5, M10, and M15 mixtures, mostly at elevated loads. This may possibly be certified to that the combinations contain a large amount of O_2_, elevated *LHV*, and small CN. These combinations of distinctive properties could deteriorate the combustion efficacy, triggering an increase in the CO level pattern^58^. Regarding UHC emission, the UHC pattern illustrates an increase with escalating engine load for the assessed mixtures, as indicated in Fig. 9d. This could be assigned to the increase in the inserted fuel with engine load, and there is no enough time to mix and combust the whole mixture, leading to an increase in UHC level. Compared to D100, the UHC level for M5, M10, and M15 mixtures grew as the methanol in the mixtures increased. The reason could be attributed to that further methanol supplement is anticipated to increase the cooling influence, triggering inadequate burning and enlarged UHC formations. These outcomes are equivalent to those described in the article^21^.

### Summarizations

**Figure 10:**
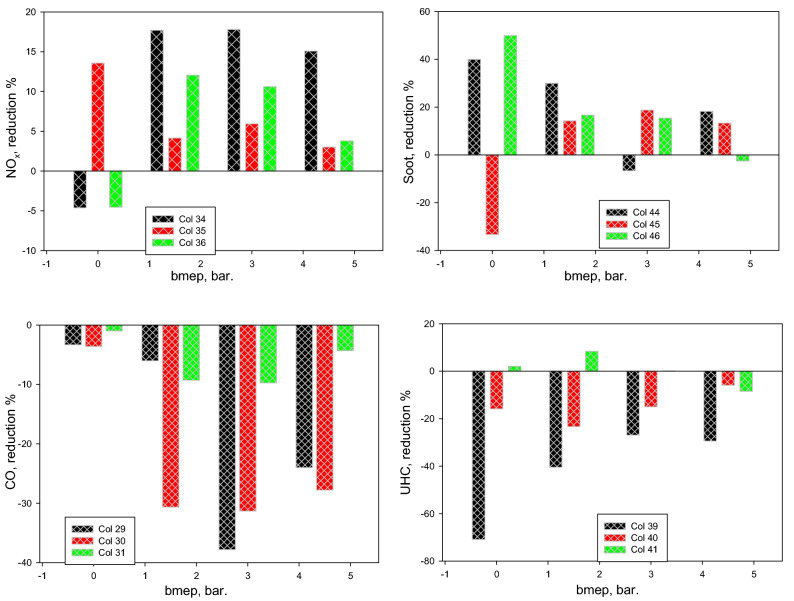
Reduction percentage of the NO_x_, soot, CO, and UHC levels with bmep for tested fuels.

**Figure 11:**
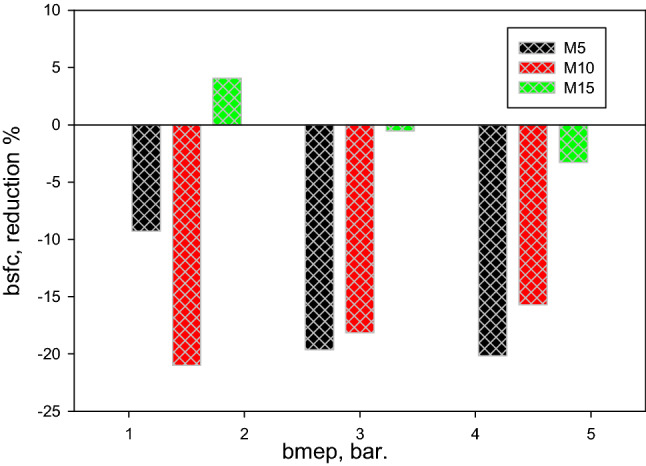
Reduction percentage of BSFC with bmep for tested fuels.

The summary of the decreased percentage in the NO_x_, soot, CO, and UHC levels of diesel-methanol blends is shown in Fig. 10. Compared to pure diesel fuel, it is recorded that the NO_x_ and soot levels dropped by an average of 15% and 30%, correspondingly. At the same time, CO and UHC increased on average by 20% and 25%, respectively. Figure 11 demonstrates the reduction percentage of the bsfc for tested fuels at various engine load. The bsfc is increased on average by 10% for diesel-methanol blends compared to diesel.

Table 5 indicates the existing study conclusions with available correlated articles. It is noticeable that the current examination signaled considerable results aiding the application of M100 in CI engines and also exhibited a reasonable association with other correlated studies.

**Table 5:** Assessment of the existing research findings with available relevant studies.

Ref	Test platform	Base fuel	Methanol segment	Cosolvent kind and quantity	Burning evaluation	Mechanical factors	Emissions
^22^	CI engine	Diesel	10% and 15% by volume	Pentanol (*C5*); 20%	Enhanced P_cyl.,_ HRR, and ID; lowered CD	Lowered BTE; enlarged bsfc	Lowered soot; enlarged NO_x_
^10^	CI engine	Diesel	20%	Microalgae biodiesel; 40%	Enhanced P_cyl.,_ HRR, CD, and ID; generated two peaks in HRR	Lowered BTE; increased bsfc	Improved soot; reduced NO_x_
^65^	CI engine	Diesel	10% and 15% by volume	–	Enhanced P_cyl.,_ HRR, CD, and ID; generated two peaks in HRR	Lowered BTE; increased bsfc	Lowered Soot; increased NO_x_
Current investigation	CI engine	Diesel	5%, 10%, and 15%	n-decanol; 20%	Lowered P_cyl._ and HRR; increased CD and ID; created two peaks in HRR	Dropped BTE; enlarged bsfc	Dropped NO_x_ and soot; boosted UHC & CO

## Conclusions

This article intended to investigate the effects of manipulating n-decanol as a cosolvent on the miscibility of M100/hydrous methanol/diesel mixtures at numerous temperatures. The tests were also expanded to evaluate the impacts of inserting n-decanol as a cosolvent with M100/diesel mixtures on engine combustion, emissions, and miscibility aspects. The three segments of methanol were 5%, 10%, and 15% combined with 20% n-decanol. The subsequent major conclusions were discovered from this investigation.

- The miscibility analysis of pure methanol/diesel and hydrous methanol/diesel mixtures implies that they are not able to mix at any segment below the assessed temperatures devoid of any outside agent. The utilization of n-decanol as a cosolvent proves a considerable capability for mixing M100 and hydrous methanol with diesel oil at various temperatures.
- There was a reduction in the *P*_*cyl.*_, d*P*/d*θ*, and HRR for M100/diesel mixtures compared with diesel oil. This is credited to the small energy content, small CN, and elevated *LHV* that trigger a worsening in the burning development. The methanol portion enlargement leads to a growth in CA50; thus, the majority of the combination is combusted in the diffusion stage. This could be clarified by the dual heights in the HRR pattern, which is associated with the quenching influence instigated by the supplement of a large portion of M100 in the combination.
- The BTE diminished, whereas the BSFC and BSEC boosted for the mixture compared with the diesel.
- There was an increase in the UHC and CO concentrations by about 60% and 50%, correspondingly, for the M5, M10, and M15 combinations comparison with diesel. The NO_*x*_ and smoke opacity intensity dropped by about 30% and 50%, correspondingly, for the combinations compared to diesel.

## Data Availability

The datasets used and/or analysed during the current study available from the corresponding author on reasonable request.

## Abbreviations

*CA*: Crank angle
*D100*: Pure diesel
*EGT*: Exhaust gas temperature
$\frac{dP}{d\theta }$: Change in pressure with crank degree
$\frac{dV}{d\theta }$: Change in in-cylinder volume with crank degree
*HRR*: Heat release rate
*M100*: Pure methanol
*M5*: 5 Vol% methanol + 20% n-decanol + 75 vol% D100
*M10*: 10 Vol% methanol + 20% n-decanol + 70 vol% D100
*M15*: 15 Vol% methanol + 20% n-decanol + 65 vol% D100
*MH10*: Hydrous methanol covering 90% methanol + 10% water
*LHV*: Latent heat of vaporization
$${NO}_{x}$$: Nitrogen oxides
*P*_*cyl*_: In-cylinder pressure
*PRR*: Pressure rise rate
